# The Association of Insulin-dextrose Treatment with Hypoglycemia in Patients with Hyperkalemia

**DOI:** 10.1038/s41598-020-79180-7

**Published:** 2020-12-16

**Authors:** Ljiljana Crnobrnja, Manogna Metlapalli, Cathy Jiang, Mauli Govinna, Andy K. H. Lim

**Affiliations:** 1 Department of General Medicine, Monash Health, 246 Clayton Road, Clayton, VIC 3168 Australia; 2 Department of Medicine, School of Clinical Sciences, Monash University, Clayton, VIC Australia

**Keywords:** Epidemiology, Endocrinology, Risk factors, Adverse effects

## Abstract

Treatment of hyperkalemia with intravenous insulin-dextrose is associated with a risk of hypoglycemia. We aimed to determine the factors associated with hypoglycemia (glucose < 3.9 mmol/L, or < 70 mg/dL) and the critical time window with the highest incidence. In a retrospective cohort study in a tertiary hospital network, we included 421 adult patients with a serum potassium ≥ 6.0 mmol/L who received insulin-dextrose treatment. The mean age was 70 years with 62% male predominance. The prevalence of diabetes was 60%, and 70% had chronic kidney disease (eGFR < 60 ml/min/1.73 m^2^). The incidence of hypoglycemia was 21%. In a multivariable logistic regression model, the factors independently associated with hypoglycemia were: body mass index (per 5 kg/m^2^, OR 0.85, 95% CI: 0.69–0.99, *P* = 0.04), eGFR < 60 mL/min/1.73 m^2^ (OR 2.47, 95% CI: 1.32–4.63, *P* = 0.005), diabetes (OR 0.57, 95% CI 0.33–0.98, *P* = 0.043), pre-treatment blood glucose (OR 0.84, 95% CI: 0.77–0.91, *P* < 0.001), and treatment in the emergency department compared to other locations (OR 2.53, 95% CI: 1.49–4.31, *P* = 0.001). Hypoglycemia occurred most frequently between 60 and 150 min, with a peak at 90 min. Understanding the factors associated with hypoglycemia and the critical window of risk is essential for the development of preventive strategies.

## Introduction

Hyperkalemia is associated with the risk of cardiac arrhythmia and cardiac arrest. Serum potassium levels above 6 mmol/L require urgent treatment to avoid cardiac instability^1,2^. Insulin-dextrose treatment (IDT) is a common first-line treatment for moderate (potassium 6 to 7 mmol/L) to severe hyperkalemia (potassium > 7 mmol/L). Unlike oral cation exchange resins which require several hours to reduce serum potassium through the gastrointestinal tract, intravenous (IV) insulin rapidly shifts potassium intracellularly^2^. Insulin stimulates the activity of the sodium-hydrogen antiporter on cell membranes, and via promoting sodium entry into cells, triggers activation of the sodium–potassium ATPase pump leading to potassium influx into cells^1^.

IDT can be complicated by hypoglycemia, which is associated with increased morbidity and mortality. The estimated incidence of hypoglycemia ranges from 6 to 21%^3–6^. The variability in estimates is likely due to differences in the population studied, clinical setting, protocol for IDT, and the definition of hypoglycemia used. Previously identified risk factors for hypoglycemia have included age, body weight, history of diabetes and pre-treatment glucose^3,7^. It was also suggested that acute kidney injury (AKI) and chronic kidney disease (CKD) may be contributing factors as well^4^. However, there is a lack of consistency on identified risk factors with some studies not confirming these findings^5,8,9^. The cause of hypoglycemia may be multifactorial and many studies have not utilized multivariable modelling to determine the significance of individual variables.

There is also limited data on the timing of hypoglycemic events or nadir of blood glucose levels following IDT, which can be dependent on the way the glucose load is given. Most institutions have guidelines on the frequency and duration of blood glucose monitoring, but most studies have not defined the optimal protocol for monitoring. While high frequency and prolonged duration of blood glucose monitoring allows hypoglycemia to be effectively detected, it should be balanced by the negative impact of excessive testing, such as patient discomfort and the need for additional resources and clinician time.

The primary aim of this study was to determine the incidence and severity of hypoglycemia following IDT for hyperkalemia, and to identify the risk factors for hypoglycemia using multivariable analysis. The secondary aim was to determine the timing of hypoglycemia and blood glucose trough following IDT as a means of evaluating the sufficiency of monitoring.

## Methods

### Study design and setting

We conducted a retrospective, multi-site cohort study at the acute hospitals within the Monash Health hospital network (Monash Medical Centre, Dandenong Hospital, Casey Hospital). Monash Health is the largest public health network located in the south-east region of the state of Victoria in Australia, servicing around one-quarter of the population of the city of Melbourne. The study population included all patients treated with IDT irrespective of admission type (day procedure, emergency, multi-day admission) or location (emergency department [ED], general ward, intensive care, operating theatre), from Jan 1, 2019 to March 1, 2020.

### Ethics approval

This study was approved by the Monash Health Human Research Ethics Committee (Monash HREC, reference number RES-20–0000-191Q) and individual patient informed consent was waived as this retrospective study used data routinely collected during clinical practice and no additional information was sought from the patients. This study was conducted in accordance with the National Health and Medical Research Council of Australia guidelines.

### Participants

All adult patients (≥ 18 years) who received IDT for a serum potassium of 6.0 mmol/L or greater were eligible. We used ICD-10 coding to find patients diagnosed with hyperkalemia. The medical records were examined to confirm IDT treatment and a pre-treatment potassium level of ≥ 6.0 mmol/L. From the eligible patients, we excluded patients with repeat admissions within the study time frame (only data from the index admission was used), missing blood glucose monitoring record, or pre-IDT hypoglycemia requiring intervention. Full baseline data was extracted, and patients were followed for the development of hypoglycemia after IDT.

### Insulin-dextrose protocol

Our local protocol required IV administration of 10 U regular insulin with 25 g of glucose as 50 mL of 50% dextrose. The alternative of 10% dextrose was rarely utilized. Our health network recommended monitoring blood glucose at 15, 30, 60, 90 and 120 min, followed by hourly checks until at least 6 h after IDT.

### Main outcomes

The primary outcome of the study was hypoglycemia, defined as a glucose level < 3.9 mmol/L (70 mg/dL) regardless of symptoms. For descriptive purposes, hypoglycemia was further characterized as level 1 (3.0 to 3.8 mmol/L), level 2 (< 3.0 mmol/L) and level 3 (functional impairment requiring assistance) as recommended by the American Diabetes Association guidelines^10^. We accepted blood glucose levels determined by either plasma or capillary (finger-prick) sampling. The secondary outcome of interest was the timing of trough glucose and hypoglycemia. The study concluded at the end of the mandatory 6 h monitoring period. For patients who received repeated IDT, the follow-up period was extended to another 6 h after the second IDT.

### Data sources and independent variables

#### Baseline factors

We used electronic medical records to determine patient demographics (age, sex and resident of aged care facility) and specific comorbidities relevant to hypoglycemia risk (diabetes, CKD, cancer, cirrhosis and excessive alcohol intake). CKD status was determined using the CKD-EPI eGFR based on IDMS-calibrated serum creatinine using a strategy recommended by Siew et al^11^. We considered an eGFR < 60 ml/min/1.73 m^2^ as consistent with significant CKD as an eGFR of 60 to 90 ml/min/1.73 m^2^ in older adults is of unclear significance. Excessive alcohol intake was defined as a chronic ingestion of ≥ 4 standard drinks daily.

#### Clinical factors

We collected data on weight (in kg) and height (in meters) and calculated the body mass index (BMI) in kg/m^2^. We used the Malnutrition Universal Screening Tool (MUST) score as a surrogate for malnutrition (a score of ≥ 2 indicates a high probability of malnutrition)^12^. The presence of sepsis was determined using SEPSIS-3 guidelines^13^ and AKI was determined using KDIGO guidelines^14^. We examined the use of specific medications within the previous 24 h which may affect the glycemic response (oral hypoglycemic agents, insulin, corticosteroids, beta-blockers, ACE inhibitors and angiotensin receptor blockers).

#### Treatment-related factors

Three main areas for treatment location were identified, namely ED, general ward and intensive care unit (ICU). We determined the insulin dose, glucose load (in grams) and dextrose concentration (in %) from medication charts in case of deviation from protocol. The use of repeat IDT within 6 h of the initial IDT was recorded, given the potential cumulative effect of insulin. As a surrogate marker of severity, we determined the highest level of intervention required to correct hypoglycemia. Although patients may have received more than one intervention for hypoglycemia, we classified the intervention into mutually exclusive categories according to a hierarchical order (observation only, food or drink, oral glucose, IV glucose bolus, and IV glucose infusion), with the highest level of intervention required defining the category of intervention.

### Statistical analysis

For descriptive analysis, we report the mean and standard deviation (SD) for normally distributed data. For significantly skewed data, we report the median and interquartile range (IQR). Between group comparisons of continuous variables were performed using Student’s t test or ANOVA. Categorical data analysis was performed using a χ^2^ test. Multivariable logistic regression was used to regress hypoglycemia on independent risk factors. Parameter estimates included the 95% confidence interval (95% CI). The initial selection of variables into the multivariable model is based on a univariable *P* < 0.10, followed by a stepwise selection process. Multicollinearity was detected by examining the variance inflation factor. Interaction terms in the logistic regression model were only considered significant at a *P* < 0.01. Multiple imputation with chained equations was used for missing data in BMI and pre-IDT glucose prior to multivariable logistic regression. The variables used to create 20 imputed datasets included age, sex, diabetes status, insulin use, use of oral hypoglycemic agents and hypoglycemia. We examined the model calibration using the Hosmer–Lemeshow test and a calibration plot, and assessed discrimination with the c-statistic. A *P* < 0.05 was considered statistically significant. Analysis was performed using STATA 16.1 (StataCorp, TX, USA).

## Results

### Patient characteristics

**Figure 1:**
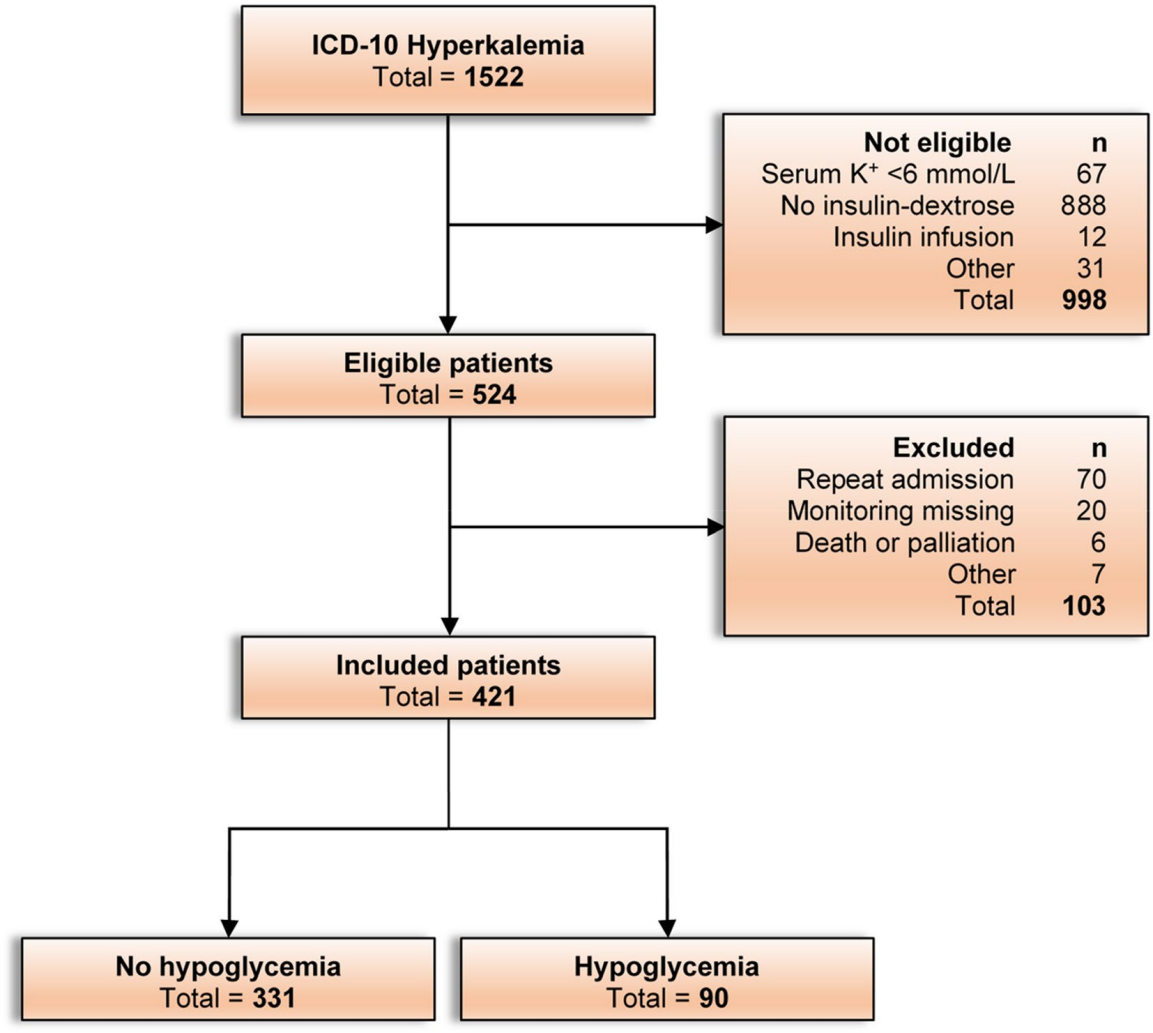
Study flow chart. Flow diagram showing the search outcomes, eligible patients, reasons for exclusion and patients included in the final analysis.

A flow chart summarizing the patient search, eligibility, exclusions, and final selection for inclusion in the study is shown in Fig. 1. The characteristics of the included patients are summarized in Table 1. On average, the cohort comprised older male patients (mean age of 69.6 years, 62.2% male), with a high prevalence of diabetes (60.3%) and CKD with an eGFR < 60 ml/min/1.73 m^2^ (70.3%). This also included 26 of 421 (6.2%) of patients with kidney failure on chronic dialysis and approximately half of all patients experienced AKI. Around one third of patients were treated with an ACE inhibitor or beta-blockers.

**Table 1:** Patient characteristics by hypoglycemia status.

Characteristic	All patients n = 421	No hypoglycemia n = 331	Hypoglycemia n = 90
Age, mean (SD), years	69.6 (15.9)	69.1 (15.3)	71.4 (18.0)
Male, n (%)	262 (62.2)	202 (61.0)	60 (66.7)
BMI,^a^ median (IQR), kg/m^2^	26.4 (23.0–32.0)	26.9 (23.6–32.4)	24.5 (21.0–29.4)
MUST score ≥ 2, n (%)	75 (17.8)	61 (18.4)	14 (15.6)
**Diabetes mellitus, n (%)**	254 (60.3)	214 (64.7)	40 (40.4)
Oral hypoglycemic agents	127 (30.2)	107 (32.3)	20 (22.2)
Insulin-requiring	100 (23.8)	87 (26.3)	13 (14.4)
eGFR < 60 mL/min/1.73m^2^,^b^ n (%)	296 (70.3)	221 (66.8)	75 (83.3)
Active cancer, n (%)	67 (15.9)	53 (16.0)	14 (15.6)
Cirrhosis, n (%)	22 (5.2)	19 (5.7)	3 (3.3)
Excess alcohol intake, n (%)	33 (7.8)	29 (8.7)	4 (4.4)
Beta-blockers, n (%)	167 (39.7)	134 (40.5)	33 (36.7)
Renin-angiotensin inhibitors, n (%)	138 (32.8)	109 (32.9)	29 (32.2)
Corticosteroids, n (%)	84 (20.0)	72 (21.8)	12 (13.3)
Sepsis, n (%)	48 (11.4)	39 (11.8)	9 (10.0)
**Acute kidney injury, n (%)**^**c**^	192 (48.6)	155 (49.5)	37 (45.1)
Stage 1	86 (21.8)	69 (22.0)	17 (20.7)
Stage 2	67 (17.0)	52 (16.6)	15 (18.3)
Stage 3	50 (12.7)	37 (11.8)	13 (15.9)

^a^Missing BMI observations, n = 11.

^b^Equivalent to KDIGO chronic kidney disease stage 3 or higher.

^c^Excluding 26 chronic dialysis patients who cannot be evaluated for AKI.

Abbreviations: MUST, Malnutrition universal screening tool.

### Insulin-dextrose treatment

Overall, more than half of patients received IDT in ED. The standard glucose load in our protocol is 25 g, which was given to most of our patients (Table 2). Only 9% (36 of 421 patients) received 12.5 g of glucose loading due to clinician discretion. Almost all patients received the glucose loading as 50 mL of 50% dextrose. Only 2% of patients received less than the standard 10 units of insulin. A repeat IDT was given to 18.5% (78 of 421 patients) patients at a mean of 186 min (SD, 95 min) after the initial treatment (Table 2). The proportion of patients receiving repeat IDT was not different between patients with hypoglycemia (18 of 90 patients) compared to those without hypoglycemia (60 of 331 patients), with a χ^2^ = 0.16 (*P* = 0.69).

**Table 2:** Details of insulin-dextrose treatment and glucose levels.

Characteristic	All patients n = 421	No hypoglycemia n = 331	Hypoglycemia n = 90
**Treatment location, n (%)**			
General ward	135 (32.1)	115 (34.7)	20 (22.2)
Intensive care	61 (14.5)	55 (16.6)	6 (6.7)
Emergency department	225 (53.4)	161 (48.6)	64 (71.1)
**Glucose quantity**^**a**^**, n (%)**			
12.5 g	36 (8.6)	28 (8.5)	8 (8.9)
25 g (standard)	382 (90.4)	300 (90.6)	82 (91.1)
50 g	3 (0.7)	0 (0)	3 (3.3)
**Short-acting insulin dose**^**b**^**, n (%)**			
5–8 Units	10 (2.4)	8 (2.4)	3 (2.2)
10 Units (standard)	407 (96.7)	322 (97.3)	85 (94.4)
12–20 Units	4 (1.0)	3 (0.9)	1 (1.1)
Pre-treatment blood glucose, median (IQR), mmol/L^c^	7.9 (6.2–11.8)	8.8 (6.6–12.0)	6.5 (5.1–8.0)
Trough blood glucose, median (IQR), mmol/L	6.0 (4.3–9.1)	7.0 (5.2–10.0)	3.0 (2.5–3.5)
Time to trough, median (IQR), min	165 (93–266)	180 (100–290)	129 (90–200)
Peak blood glucose, median (IQR), mmol/L	10.0 (7.3)	10.9 (7.6–15.5)	8.3 (5.6–10.5)
Time to peak, median (IQR), min	105 (40–240)	90 (37–227)	160 (40–290)
Trough blood glucose 3.0 to 3.8 mmol/L, n (%)	45 (10.7)	N/A	45 (50.0)
Trough blood glucose < 3.0 mmol/L, n (%)	45 (10.7)	N/A	45 (50.0)
Repeat treatment < 6 h, n (%)	78 (18.5)	60 (18.1)	18 (20.0)

^a^Two patients received 10% dextrose rather than 50% dextrose.

^b^Six patients received Novorapid 10 Units, rather than Actrapid 10 Units.

^c^Missing observations in pre-treatment blood glucose (n = 22).

### Hyperkalemia

The median pre-treatment potassium was 6.4 mmol/L (IQR, 6.2 to 6.8 mmol/L). The change in serum potassium post-treatment was normally distributed, with a mean of 1.4 mmol/L (SD, 0.8 mmol/L) lower than pre-treatment. The use of oral sodium polystyrene sulfonate for potassium lowering was not different between patients with hypoglycemia (65 of 90 patients) and without (204 of 331 patients) hypoglycemia (χ^2^ = 3.44, *P* = 0.07). The use of bicarbonate (IV or oral) was not different between patients with hypoglycemia (9 of 90 patients) and without (30 of 331 patients) hypoglycemia (χ^2^ = 0.07, *P* = 0.79). Salbutamol use for lowering potassium was also similar between patients with hypoglycemia (8 of 90 patients) and without (41 of 331 patients) hypoglycemia (χ^2^ = 0.84, *P* = 0.36). Thus, the use of adjunct potassium lowering treatment was equally distributed between the two groups based on hypoglycemia outcome. Finally, the degree of potassium lowering was not different based on hypoglycemia status, with a mean difference of 0.17 mmol/L (95% CI: -0.03 to 0.37 mmol/L, *P* = 0.09).

### Hypoglycemia

The incidence of hypoglycemia was 21.4% (90 of 421 patients), with 50% (45 of 90 patients) of these representing severe (level 2) hypoglycemia (Table 2). We could not assign patients to level 3 hypoglycemia as symptoms and functional significance of hypoglycemia were not consistently documented in the medical records. For the management of hypoglycemia, 9% (8 of 90 patients) received food and drinks only, 20% (18 of 90 patients) received oral glucose, 38% (34 of 90 patients) received a single bolus of IV 50% dextrose, and 6% (5 of 90 patients) required a dextrose infusion. Two patients received IM glucagon. There was 26% (23 of 90 patients) who did not have specific management of hypoglycemia (mean trough glucose, 3.4 mmol/L; SD, 0.6 mmol/L).

### Risk factors for hypoglycemia

**Figure 2:**
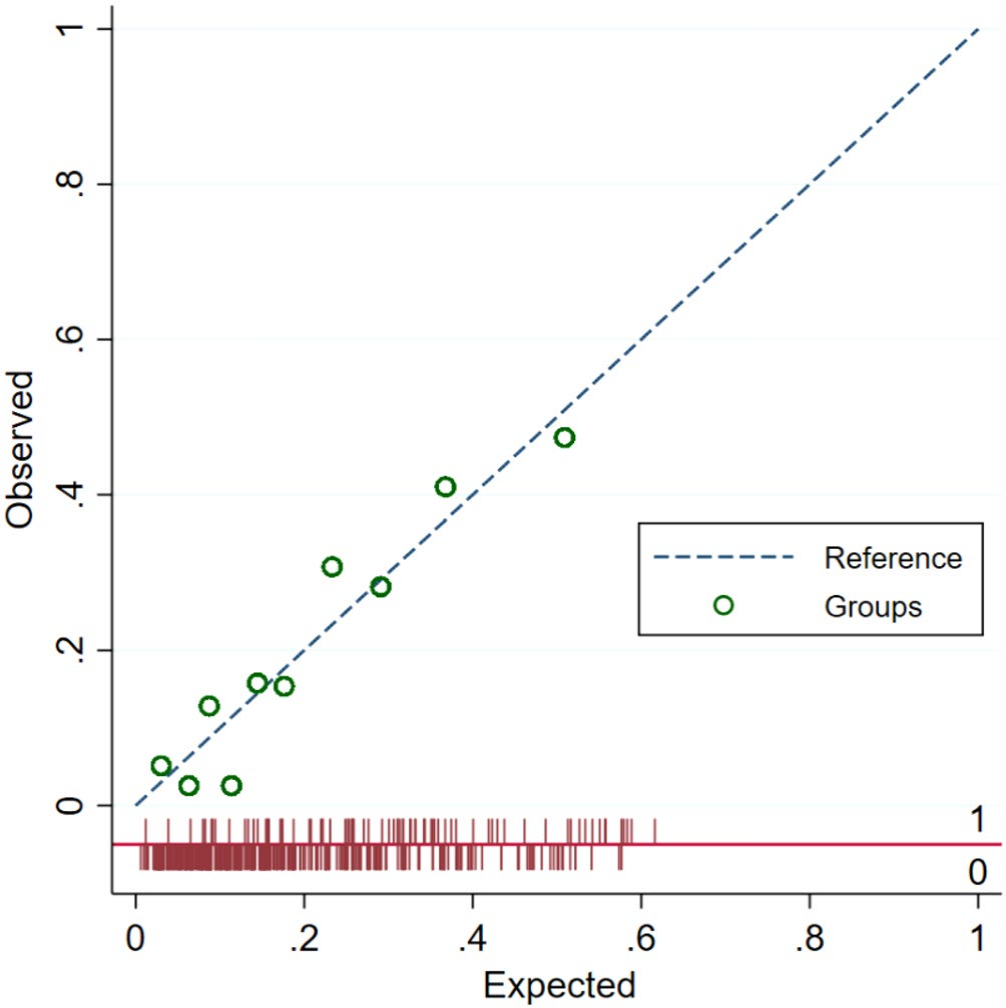
Calibration plot of multi-variable logistic regression model. Model diagnostics to determine how well the model fits the data. Perfect alignment along the dotted line indicates perfect correlation between predicted and observed outcomes.

The results of the logistic regression analysis are shown in Table 3. In the univariable analysis, the identified risk factors for hypoglycemia were BMI, diabetes, eGFR < 60 ml/min/1.73 m^2^, insulin treatment, pre-treatment glucose, and location of treatment in ED. In the selection of the variables for multivariable analysis, there was weak evidence of multicollinearity between diabetes status and insulin treatment (*r* = 0.49; variance inflation factor = 1.32). These variables were moderately correlated and insulin treatment was not independently associated with hypoglycemia (OR 0.80, 95% CI: 0.37 to 1.71, *P* = 0.56) when diabetes status was included in the multivariable model. Similarly, the use of oral hypoglycemic agents was not significant after accounting for diabetes (OR 0.93, 95% CI: 0.46 to 1.88, *P* = 0.84). No significant statistical interaction was noted in the final multivariable model. The c-statistic of the multivariable model was 0.76. The model calibration was satisfactory (Hosmer–Lemeshow, χ^2^ = 7.24, *P* = 0.51), as seen in the calibration plot (Fig. 2).

**Table 3:** Logistic regression analysis.

Variable	Univariable		Multivariable ^d^	
	OR (95% CI)	*P* value	OR (95% CI)	*P* value
Age	1.01 (0.99–1.02)	0.230		
Female sex	0.78 (0.48–1.28)	0.328		
BMI (per 5 kg/m^2^)^a^	0.82 (0.68–0.97)	0.024	0.83 (0.69–0.99)	0.048
MUST score ≥ 2	0.82 (0.43–1.54)	0.528		
Diabetes mellitus	0.44 (0.27–0.70)	0.001	0.57 (0.33–0.98)	0.043
eGFR < 60 ml/min/1.73m^2^	2.49 (1.37–4.53)	0.003	2.94 (1.56–5.54)	0.001
Cirrhosis	0.57 (0.16–1.96)	0.369		
Active malignancy	0.97 (0.51–1.83)	0.916		
Excess alcohol intake	0.48 (0.17–1.42)	0.185		
Oral hypoglycemic medication	0.60 (0.35–1.03)	0.066		
Regular insulin treatment	0.47 (0.25–0.89)	0.021		
Beta-blockers	0.85 (0.53–1.38)	0.512		
Renin-angiotensin inhibitors	0.97 (0.59–1.59)	0.899		
Corticosteroids	0.55 (0.29–1.07)	0.080		
Sepsis	0.83 (0.39–1.79)	0.637		
Severe acute kidney injury^b^	1.50 (0.78–2.86)	0.221		
Pre-treatment blood glucose^c^	0.84 (0.78–0.91)	< 0.001	0.88 (0.81–0.95)	0.002
Repeat treatment < 6 h	1.13 (0.63–2.03)	0.685		
Glucose load 12.5 g	1.05 (0.46–2.40)	0.897		
Treated in Emergency Department	2.60 (1.57–4.30)	< 0.001	2.53 (1.49–4.31)	0.001

^a^Missing observations in BMI (n = 11).

^b^Stage 3 AKI and excluding long-term dialysis patients (n = 395).

^c^Missing observations in pre-treatment blood glucose (n = 22).

^d^Multiple imputation using chained equations (m = 20) for missing observations.

### Timing of hypoglycemia

**Figure 3:**
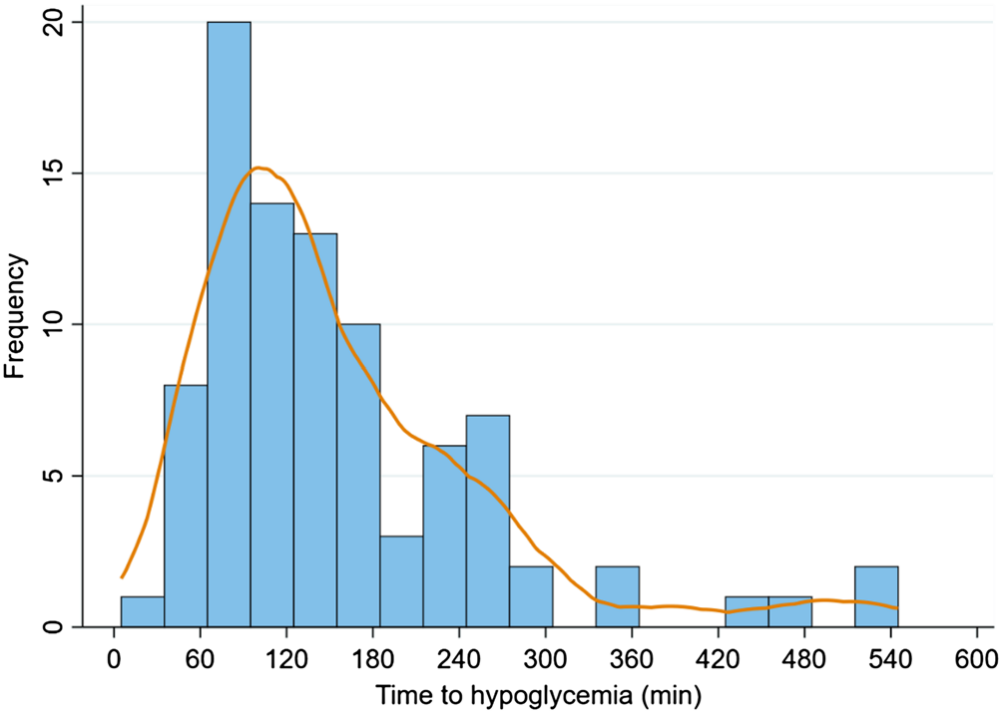
Timing of hypoglycemia after insulin-dextrose treatment (n = 90). Kernal density line (dark orange) highlights the peak occurrence of hypoglycemia at around 90 min after treatment was given. Additional hypoglycemia events occurred after the mandatory 6 h monitoring in patients who received a second treatment. Only two patients who received a single treatment experienced hypoglycemia after 5 h.

Figure 3 demonstrates the timing of hypoglycemia episodes. Most occurred in the second hour of treatment and peaked at 90 min. Hypoglycemia was rare before 30 min and rare after 5 h, unless a repeat IDT was given. The time frame or window with the highest frequency of hypoglycemia was between 60 and 150 min. After a single treatment, the trough glucose occurred much earlier in patients who experience hypoglycemia compared to those who did not, at a mean of 142 min (SD, 74 min) compared to 179 min (SD, 101 min). The difference of 37 min (95% CI: 14 to 60 min) was statistically significant (*P* = 0.002).

## Discussion

In this study, the incidence of hypoglycemia was 21% and half were level 2 in severity. In the larger studies, the incidence of hypoglycemia following IDT ranged from 13 to 21%^3,4,6,7,9^. Our result is concordant with these estimates. However, the risk factors for hypoglycemia identified previously have not been consistent^4,6,15–17^. Few studies have used a multivariable model to account for these potential risk factors. Hence, we have taken such an approach, and noted that some of these risk factors were not independent of each other in predicting hypoglycemia. We identified the independent predictors of hypoglycemia as diabetes status, pre-IDT glucose, BMI, eGFR < 60 ml/min/1.73 m^2^ and treatment location in ED.

The pre-IDT blood glucose level is a reliable predictor of hypoglycemia which is consistent across many studies. Various cut-off values have been used to predict a higher risk of hypoglycemia, ranging from 4.7 mmol/L to 7.8 mmol/L^5–7,18^. In our cohort, every 1 mmol/L increase in pre-IDT glucose was associated with a 12% lower odds of hypoglycemia, after allowing for diabetes and the other covariates. We chose to model pre-IDT blood glucose as a continuous rather than binary variable given the wide-range of cut-off values defining risk previously reported. In the univariable analysis, diabetes status was associated with a lower odds of hypoglycemia. Diabetes has been shown to be protective in other studies^5,6,15^. However, after adjusting for pre-IDT blood glucose in the multivariable model, the effect of diabetes status on hypoglycemia risk was attenuated.

The effect of BMI on hypoglycemia risk merits consideration. We showed that on average, an increase in BMI of 5 kg/m^2^ was associated with a 17% lower odds of hypoglycemia. Not all previous studies have analyzed body weight but there is evidence supporting this association. Boughton et al. reported that patients who experienced hypoglycemia were on average 15 kg lighter than patients who did not have hypoglycemia^3^. Schafer et al. noted an ordinal relationship between a lower body weight and the severity of hypoglycemia^4^. Malnutrition is a known risk factor for hypoglycemia in hospitalized medical patients irrespective of diabetes status^19^. In our study, a high MUST score was not associated with hypoglycemia. However, the MUST score is a screening tool which has not been studied in the context of IDT. Furthermore, the MUST was completed on admission. Patients located outside ED may have already been treated for malnutrition at the time of IDT. While we do not believe the MUST score predicts hypoglycemia risk following IDT, it should be verified in other studies. It is also possible that other tools assessing malnutrition may reveal an association.

In advanced CKD, insulin clearance drops dramatically, concurrent with impairment of hepatic insulin metabolism and reduced gluconeogenesis^20,21^. In epidemiological studies, patients with diabetes and an eGFR < 60 ml/min/1.73 m^2^ have reduced insulin requirements^22^. A large study of 243,222 patients and over 2 million capillary glucose measurements demonstrated that an eGFR < 60 ml/min/1.73 m^2^ is a risk factor for hypoglycemia, both in patients with and without diabetes^23^. In our study, an eGFR < 60 ml/min/1.73 m^2^ was associated with a 2.9-fold higher odds of hypoglycemia compared to patients with a preserved eGFR, after adjusting for diabetes status and other covariates. However, there is conflicting evidence. While some studies concur that CKD is a risk factor for hypoglycemia, others do not. The positive studies have assessed risk in patients with end-stage kidney disease or a serum creatinine over 220 µmol/L^4,6,7^. Among the negative studies, two did not explicitly define CKD, one used a cut-off eGFR of 60 ml/min/1.73 m^2^, and another looked at end-stage kidney disease^3,5,9,18^. Thus, one possible reason for the heterogenous results is how CKD is defined. Another confounding matter is concurrent AKI. In our analysis, stage 3 AKI was not associated with hypoglycemia risk. While there is good data that AKI is a risk factor for subsequent hypoglycemia in patients with diabetes^24^, such evidence is lacking with IDT. Insulin resistance is also common in AKI due to increased proinflammatory cytokines and counterregulatory hormones^25^, which may offset hypoglycemia risk from reduced insulin clearance.

We noted a higher rate of hypoglycemia in ED compared to the general wards or ICU. The reason was unclear, and we can only postulate the cause. One possibility is the periprocedural fasting often observed in ED presentations. Poor oral intake from illness combined with waiting room delays could also contribute. Studies are needed to confirm this association. Then, further research is recommended to determine if prolonged fasting or poor oral intake are contributory factors. Such patients may benefit from glucose infusions rather than bolus glucose per standard IDT.

We could not demonstrate that oral hypoglycemic agents or regular insulin use were associated with a higher risk of hypoglycemia. In contrary, the odds of hypoglycemia were lower. As a retrospective study, it was not always possible to determine the timing of the last dose of glucose lowering agents. We included the use of glucose lowering agents within 24 h prior to IDT, but a specific time or omission of medications could not be established. Medication omissions occurred in 38% of ED presentations^26^. Unwell patients may not have tolerated their regular medications or self-omitted them. Ultimately, this variable may have served as a surrogate marker for diabetes, and the lower odds of hypoglycemia reflected diabetes status and pre-IDT glucose. A similar paradox was observed by Apel et al., who reported that patients with diabetes not receiving glucose lowering agents prior to admission had higher odds of having a hypoglycemic event^7^. In multivariable analysis, glucose lowering agents were not independently associated with hypoglycemia.

There is an association between RAS blockers and hypoglycemia in patients with diabetes^27^, and between beta-blockers and hypoglycemia in hospitalized patients with diabetes acutely managed with insulin^28^. Beta-blockers reduce hepatic gluconeogenesis and reduce glycogenolysis but the mechanism of hypoglycemia with RAS blockers is unknown. There is limited data from IDT studies on the effect of these medications on hypoglycemia. We did not detect any association in our study and similarly, Coca et al. did not find a significant effect of RAS blockers or beta blockers on hypoglycemia^5^. Although one previous study found that salbutamol use (within 1 h of IDT) and corticosteroid use (within 2 h of IDT) were protective from hypoglycemia^6^, we only found weak evidence that corticosteroids was protective and there was no effect of salbutamol. It is possible we did not detect these associations due to an issue of medication omission.

Blood glucose monitoring practices vary and there is limited data defining the window of greatest risk. In several studies, hypoglycemia tends to occur between 1 and 3 h after IDT^6,7,29^. We confirmed that the critical period is between 60 to 150 min, with a peak at 90 min. Thus, the higher frequency of monitoring during this window is justified. In determining the frequency of blood glucose checks, clinicians should balance hypoglycemia risk with patient discomfort and staff workload. Our data suggests that omitting the glucose check at 15 min is reasonable in patients with low risk. Hypoglycemia was rare after 5 h unless repeat IDT was given. Only 3% of hypoglycemia episodes occurred in the last hour. While hypoglycemia events have been described as long as 7.5 h after IDT^8^, our data is comparable with Tran et al., who showed that only 8% of hypoglycemic events occurred between 3 and 6 h^6^. These results suggest that glucose monitoring for 6 h is adequate. Finally, we did not detect an increased risk of hypoglycemia with repeat IDT. However, repeat IDT was given after an average of 184 min following the first IDT, which is after the critical window. Furthermore, Tran et al. showed that the odds of hypoglycemia was not increased if the glucose level was > 7.8 mmol/L prior to retreatment^6^.

### Strengths and limitations

We conducted a multisite study with broad representation of patients and treatment location, which improves the generalizability of our results. We looked at multiple blood glucose readings after IDT and were able to establish an accurate incidence of hypoglycemia and reliably determine the critical window for hypoglycemia. Unlike many previous studies, we included the major confounding factors in a multivariable analysis to reduce the possible biases associated with observational studies. However, as an observational study, we cannot infer causation. There was also missing data on pre-IDT glucose readings and BMI which required the use of multiple imputation, and we have assumed these data were missing at random. As missing data only represented 3% and 5% of available BMI and pre-IDT glucose observations respectively, we believe the risk of bias is minimal. There was an overall poor documentation of hypoglycemia symptoms to assess severity which may have underestimated our severity assessment.

### Clinical implications

It is not clear which is the best approach to reduce hypoglycemia risk. Insulin dose reduction has been suggested in patients with advanced CKD^5,7,8,30,31^. Two studies have compared hypoglycemia incidence in patients with CKD receiving 5 U versus 10 U of insulin. Pierce et al. found no difference in hypoglycemia^8^ but Larue et al. reported a 9.1% difference in the incidence of hypoglycemia and comparable potassium lowering^31^. Given the effect of BMI of hypoglycemia risk, a weight-based approach may be worthwhile. Wheeler et al. compared weight-based insulin dosing (0.1 units/kg to a maximum of 10 units) with routine use of 10 U insulin, and reported a difference in hypoglycemia incidence of 15.2% favoring the weight-based strategy^32^. A glucose top-up between 60 and 90 min is also a reasonable approach, as suggested by Apel^7^. Finally, we could also start with a higher glucose load in high risk patients, such as 50 g, as recommended by a meta-analysis of IDT studies^33^.

### Conclusion

The incidence of hypoglycemia remains relatively high with a standard IDT protocol of 25 g of glucose and 10 U of insulin. Increased vigilance is needed for patients with an eGFR < 60 ml/min/1.73 m^2^ or low BMI, and additional measures to avoid hypoglycemia may be considered. Further studies are required to determine if the better option is to provide a second glucose load at the time of peak hypoglycemia, or to reduce the dose of IV insulin. Studies are needed to understand why patients treated in ED have a higher incidence of hypoglycemia compared to other locations.

## Data Availability

The datasets generated during and analyzed during the current study are not publicly available due but are available from the corresponding author on reasonable request, subject to approval by the Monash Health research directorate.
